# Adding Heart Disease Outcomes to REGARDS: The REGARDS Myocardial Infarction Ancillary Study

**DOI:** 10.1093/geroni/igaa057.3154

**Published:** 2020-12-16

**Authors:** Raegan Durant, Emily Levitan, Paul Muntner, Todd Brown, Monika M Safford

**Affiliations:** 1 University of Alabama at Birmingham, BIRMINGHAM, Alabama, United States; 2 University of Alabama at Birmingham, Birmingham, Alabama, United States; 3 Weill Cornell Medical College, New York, New York, United States

## Abstract

The REGARDS-MI ancillary study provided new outcomes of heart disease events and adjudicated cause of death. A primary focus has been disparities in and risk factors for coronary artery disease. We demonstrated that compared to White men, Black men have a higher risk of fatal coronary heart disease (CHD) but a lower risk of non-fatal CHD. Ongoing work is investigating potential reasons for this. We have investigated the role of CHD in aging including the relationship between heart failure and cognitive function and the association of MI with functional status. The REGARDS-MI study has served as a platform for mentoring trainees and early stage investigators, many from underrepresented groups, and provided data to a large number of investigators to purse research in CHD. To date, REGARDS-MI has contributed to nearly 200 publications and spawned additional ancillary studies. This presentation will highlight some of these publications and other research in progress.

